# Identifying a probable suicide cluster in an acute care psychiatric hospital in the Eastern Cape, South Africa

**DOI:** 10.4102/sajpsychiatry.v27i0.1646

**Published:** 2021-04-19

**Authors:** Ruwayda Jacobs, Christoffel Grobler, Johanita Strumpher

**Affiliations:** 1 Department of Emergency Medical Care, Faculty of Health Sciences, Nelson Mandela University, Port Elizabeth, South Africa; 2 Department of School of Health Systems and Public Health, Faculty of Health Sciences, University of Pretoria, Pretoria, South Africa; 3 Department of Nursing, Faculty of Health Sciences, Nelson Mandela University, Port Elizabeth, South Africa

**Keywords:** patient suicide, psychiatric hospital, suicide clustering, mental health team, psychiatric nurses

## Abstract

**Background:**

Two young male patients who were diagnosed with drug-induced psychosis committed suicide in a psychiatric hospital in South Africa within a month of each other. The psychiatric nurses working in the hospital had never before had to deal with a similar trauma of suicide cluster.

**Aim:**

To assess the psychiatric nurses’ experiences of suicide cluster in an inpatient psychiatric setting.

**Setting:**

A psychiatric hospital in the Eastern Cape, South Africa.

**Method:**

A qualitative design was used. The research population consisted of psychiatric nurses who were purposively selected. Data were gathered using in-depth interviews where the participants narrated their experiences of the incidents. The interviews were transcribed verbatim and the data was coded using descriptive and explanatory codes. Trustworthiness was ensured. Ethical principles of justice, autonomy, beneficence and non-maleficence were ensured.

**Results:**

An attempt was made to compare the suicides of two patients with the characteristics of cluster suicide to determine if clustering took place. Two young males committed suicide in an institutional setting within a month of each other. Other characteristics present included using the same method, in the same venue and in the same unit. They had similar educational and social backgrounds. The second victim knew the first victim and was aware of what happened.

**Conclusion:**

When the two events are analysed together it would seem as if clustering did occur. The suicide victims knew each other and victim number 2 was aware of the facts of the first suicide. They were in their early 20’s, were mentally ill and committed suicide in the same site, using the same method and were both institutionalised at the time. Members of the mental healthcare team should be made aware of the characteristics of clustering so that when a suicide attempt occurs in a place where mentally-ill individuals are cared for, measures can be put in place to prevent another patient from copying such an event.

## Introduction and background

Clustering has been described as an excessive number of suicides or suicidal attempts, usually two or more completed or attempted suicides, occurring in close temporal, geographic or interpersonal proximity of each other than would normally be expected in a given community.^1,2^ A phenomenon of great concern to those who work with adolescents or young adults is the occurrence of suicidal clusters where a young individual may attempt or complete suicide after the death because of accident or suicide of a person whom the victim is trying to emulate.^3,4^

Headspace^5^ differentiates between point clusters and mass clusters. Point clusters involve suicides that are close in time and geographical area such as those that occur in institutional settings such as hospitals. A mass cluster involves suicides that occur in a certain time span and does not have much to do with geography as can be demonstrated by suicides associated with celebrities.^4,5,6^

According to Poland et al.^7^ the mechanism behind clustering may be contagion where the message is transmitted through personal communication or mass media describing a suicide. Sarkhel,^2^ Kõlves and Yu Wen Koo^6^ refer to the term contagion as imitation which focuses on a stimulus-response process to explain suicide clusters. A vulnerable individual may have accessed the idea of suicide from someone else, and decided to act upon it.^8^ There is an increased risk for suicide clusters to develop in hospitalised or institutionalised young people than those living in the community.^3^ In all these situations, the second person to commit suicide and those that follow were aware of what happened to the first person in the cluster, although they may not have had detailed knowledge of the event. It is not clear what triggers cluster formation, or what causes it to continue or eventually subside. Clustering may start with an individual who is already vulnerable and who may be socially connected to another person who has died, usually not because of natural causes but the suicide of a peer. According to Sarkhel^2^ contagion refers to a process by which exposure to suicidal thoughts, behaviours or death influences others to attempt or commit suicide. It may occur because of individuals passing information, or the idea of suicide between themselves. Exposure may occur when the mass media publish information on the suicide such as the suicidal death of a celebrity. It may also occur when an already troubled teenager is exposed to an unnatural death.

The person may make the discovery of a completed suicide or even the accidental death of a peer, and decides to imitate it.^2,9^

### Problem

The facility in question is the admission unit of a state psychiatric hospital in South Africa where patients who are seriously ill with acute mental disorders are treated. Most of the patients are diagnosed with schizophrenia, mood disorders, personality disorders, substance abuse and substance induced psychosis. These patients are acutely ill and may present with behavioural disturbances such as physical and verbal aggression, restlessness and agitation. On admission most patients are treated in closed units until the acute symptoms subside. In 2012, in rapid succession two young male patients, who were being treated in the same unit, committed suicide by hanging themselves in the same space.

Patient number one was a 21-year-old male from a lower socio-economic background, belonging to the Muslim faith. He had a long history of drug abuse (cannabis, mandrax and methamphetamine) and had been admitted on numerous occasions with a diagnosis of drug induced psychosis. At the time of the suicide he had been in hospital for 7 days. His condition had been stabilised and he was exhibiting the characteristics of an outgoing, friendly personality. His family was dysfunctional and he did not get along with them. Although the patient apparently felt rejected by his family, he demanded contact with them, requesting their visits. On the day of his death, he contacted them telephonically. During the conversation, the family member apparently refused to visit. On re-joining the group of patients, he participated in activities with apparent joy, listening to music and dancing; appearing carefree. Shortly afterwards the patients left to go for lunch. He left the dining room, collected a bed-sheet and hung himself in the toilet, where he was discovered shortly afterwards.

Patient number two was a 25 years old male of the Roman Catholic faith, from a similar socio-economic background and was well acquainted with patient number one as they were often treated in the hospital at the same time. He also had a diagnosis of drug induced psychosis because of alcohol abuse, and the use of cannabis and mandrax. He had been an inpatient at the time of patient number one’s death but had been discharged soon after the event. He was re-admitted about 2 weeks later. On admission he was psychotic, restless as well as verbally and physically aggressive, and was unable to socialise with the patients in the unit. Because of his behavior he was secluded earlier that day. During change-over, as the day staff was leaving the unit, he kept shouting at them, trying to convince one of them to help him phone home. When nobody would help him, he shouted a veiled threat that ‘… you will be sorry. Watch what I will do … nobody cares about me’. Whilst the night staff was involved in the change-over and began organising the night-time activities, he went into the same toilet as patient number one and hung himself.

His body was discovered soon afterwards.

Before these incidents, hospital staff had very little exposure to patient suicide. Some patients, known to staff, did commit suicide, but this occurred outside the hospital. Suicide attempts are made from time to time in psychiatric hospitals, but no previous patient had died by committing suicide in this hospital. As the two events occurred only a month apart, the staff members as well as the researchers considered the possibility of cluster formation.

The early detection of a suicide cluster is useful as it may help gatekeepers working in institutions, such as psychiatric hospitals; identify the potential of one patient copying another in committing suicide. The aim of this article was to investigate the data of the two completed suicides to determine if cluster-formation took place so that a protocol can be developed to help staff in mental health settings to recognise the risks for vulnerable youth and put prevention strategies in place.

## Method

A qualitative, explorative, descriptive and contextual study was done to determine the psychiatric nurses’ experiences of the two events. Although all staff members in the hospital had been traumatised by the events it was decided to focus the study on the psychiatric nurses as they were a homogeneous group and had maximum contact with the patients. There had been nine psychiatric nurses who had worked in the unit on both occasions and they were invited to participate in the interviews. Not all the nurses meeting the inclusion criteria participated in the study as some were doing night duty, one was on leave and two chose not to participate as they still found it difficult to talk about their experiences. All the participants ranged in age between 30 and 50 years. Four nurses were male whilst the rest were female. All of the nurses had been working in a psychiatric facility for at least 5 years.

Data were collected by conducting in-depth semi-structured interviews with the participants. Each interview lasting between 25 min and 45 min, were audio-recorded and then transcribed verbatim. Data gathering continued until data saturation occurred. Collateral data were obtained from the patients’ files as well as an incident report that had been compiled for management by the staff that were present at time of the incidents. Content analysis was done and three themes related to the experiences of the nurses were identified. One of the themes described the similarities between the two suicidal incidences and made the researchers wonder if cluster formation took place. In this article, this theme will be described.

Trustworthiness was ensured by member checking; asking the participants to read the transcribed interviews to ensure that it told the whole story; peer review by asking psychiatric nurses as well as mental health professionals to assess the validity of the themes and recommendations; triangulation by using multiple sources of information such as interviewing and keeping a reflective journal; doing a pilot interview and ensuring a rich description of the methodology as well as the findings.^10^

### Ethical considerations

A high ethical standard was ensured by getting permission for the study from the local university’s Research Ethics Committee (H14-HEA-NUR-007), the provincial Department of Health, the hospital’s Research Committee, the acting hospital manager as well as the nursing service manager. Participants were asked to give informed consent after being told that privacy and confidentiality will be ensured and that their information will be reported anonymously. Although participants were reassured that they could withdraw from the study at any time, nobody made use of this offer. Care was taken to ensure that although reference will be made to the two particular patients, it would not be possible to identify them.^11^

## Results

Authors associate different characteristics to describe cluster suicide. The criteria identified by various authors as described in the literature review will be used to identify if clustering took place.

The two suicides can be described as *a point cluster*^5^ as they occurred close in *time* and had *geographical proximity*. The second suicide occurred a month after the first one and imitated the first suicide in venue and method. Both victims were *institutionalised* at the time of their death. The criteria for the *number* of victims were met as more than one death occurred.

*Contagion* did occur as the second patient *imitated* the first one. ‘They were in the ward at the same time when the (first) suicide happened I think he saw that as a way out’. As the second patient was being treated in the unit at the time of patient number one’s death, he was *familiar* with what had happened. ‘The second guy was in the ward when that guy (first victim) committed suicide. He was discharged but he came back’.

It was also known that the two victims had an *interpersonal relationship* as a result of being inpatients in the unit at the same time on more than one occasion. ‘They were very good friends … they were best buddies … He was close to the first patient’.

With both patients the *trigger* seems to have been a breakdown in family relationships. Patient number one was able to make contact with his family but was apparently rejected. ‘Because he was missing the family and the family did not come to visit’. Patient number two was not allowed to phone because of ward policy, which did not have a provision for telephone calls being made late in the afternoon, but he was aware that his family was angry with him. ‘I think it was due to frustration and lack of family support and rejection … did not have family support’.

The *demographic information* and background of the two patients were similar. They were both males in their early twenties, were from a coloured race, had experienced poverty, did not complete their school education and were unemployed at the time of death. Both seemed to be *disenfranchised* as they were drug abusers: ‘He was a drug addict. The family … dealt drugs, prostitution. So he did not have a good background’. Their abuse of substances and defaulting on treatment caused repeated admission to the hospital. ‘At the time he had more than three admissions, I think with the same diagnosis of substance induced psychosis’. On admission, their symptoms were similar, with similar emotions being expressed namely, an inability to control their anger: ‘That evening he was shaking the gates … he is having this underlying aggression’.

## Discussion

It is not clear what triggers the cluster formation, or what causes it to continue and eventually subside. To identify if clustering took place, one should focus on what happened after the first suicide. Jones et al.^4^ described that individuals may be socially connected through shared characteristics. Gould, Wallenstein and Davidson^9^ tried to identify criteria which may be used to identify individuals at risk for the possibility of forming a suicide cluster. These criteria include features such as the presence of mental illness, history of substance abuse, history of previous attempts, recent loss, family problems including instability, dysfunction or violence, an abnormal response to stress, a psychophysiological predisposition and the likelihood that an individual is more vulnerable to incorporating ideas or acts of suicide into their concept of self.

The two patients shared characteristics such as age, gender, race, diagnosis of being mentally ill (substance induced psychosis), a history of substance abuse, a limited ability to handle stress, adverse social circumstances, having relationship problems with their dysfunctional families and being institutionalised which may all have contributed to their vulnerability. Patient number one had a history of previous suicidal attempts.

The hypothesis a number of authors put forward that vulnerable individuals may be influenced by the death of a peer^2,7^ does not support the theory of contagion, but stresses that the individuals are probably imitating what happened during the first death. He states that individuals with shared characteristics may be more vulnerable and more easily influenced by the actions of a peer. Exposure may also occur when an individual has been exposed to death such as making the discovery of a completed suicide, or even the accidental death of a peer. The second patient did view the first incident after the death had been discovered. Imitation occurs when an individual who is already troubled becomes aware of the way a suicide occurred and decides to imitate it.^12^ Patient number two had knowledge about the first patient’s suicide which may have contributed to his choice of method and venue. He may have identified with patient number one and decided to implement the same solution to his problems.

### Recommendations

Hawton et al.^1^ describe five phases in preventing a cluster from being formed namely preparation, identifying possible cluster formation, responding, stepping down the response, and evaluating and modifying the intervention plan accordingly. To prevent suicide clusters the mental health team must try and prevent the first suicide from occurring. The problem with trying to prevent suicide is that the patient’s actions may stem from careful planning or from an impulse, both of which may not be predictable. It will be wise to remember that it is in the patient’s best interests to keep the plan secret to ensure success. The mental health team must find a balance between being proactive and not overreacting, which can trigger the suicidal act.^1^ Should a suicide occur, they should be vigilant and determine the risk of any other individual in the same institution from imitating the suicidal behaviour.

In phase one, the preparation phase, a multi-professional suicide prevention team can develop action plans, review and respond to any suspicious behaviour.^1^ The team members as well as hospital staff should receive training including suicide awareness, an understanding of suicide clusters and cluster formation; identification of warning signs, suicide reduction strategies; bereavement support and awareness of normal and abnormal responses to trauma.^1,13^

In phase two, the identification phase, staff must be vigilant and identify vulnerable young people who may be at risk of imitating a suicidal death. Mental health team members must be able to assess suicidal ideation on admission, as well as during the patient’s stay.^14^ Staff members should be mindful that their assessment may not be enough, or may be based on inadequate information regarding the patient: as such, another mental health professional, for example a psychologist who may have identified suicidal risk in a patient on admission, should be encouraged to share their concerns with nursing staff. In fact, all members of the mental health team should alert each other of a possible suicidal risk of a patient.^1,14^

In phase three, the responding phase, all patients in a facility should be deemed to be at risk, especially the ones who meet the risk factors. The normal suicide prevention activities should be implemented. Staff must ensure good relationships with patients, not rely on no-suicide contracts or 15-min checks. There must be close cooperation and good communication between team members where they inform each other about observations or any changes in a patient’s circumstances such as a crisis occurring outside the hospital. Staff should respond to the support needs of all inpatients and facilitate the necessary treatment. Patients should not be given leave of absence and should only be discharged if they are definitely not at risk anymore.^1,13^

The team should immediately respond to any perceived or real emergency. It is also important for the team members to receive sufficient support. This may include debriefing and supporting the person making the discovery and the first responder.^1,15^

In phase four, as suggested by Hawton et al.,^1^ the team should make a decision to withdraw when the danger of more suicides in the cluster has passed. In the last phase, phase five, the team will review their actions and decide on how to react in future to prevent clustering from occurring.

## Conclusion

Patients admitted to mental health facilities can be seen as individuals who are already vulnerable. When this person is exposed to the trauma of a fellow patient committing suicide in the facility it may be very difficult to handle the resultant stress. This may be the trigger that leads to cluster formation. This means that the staff in mental health facilities should be vigilant to prevent patients from committing suicide.
